# Aortic Stiffness: Epidemiology, Risk Factors, and Relevant Biomarkers

**DOI:** 10.3389/fcvm.2021.709396

**Published:** 2021-11-08

**Authors:** Rebecca Angoff, Ramya C. Mosarla, Connie W. Tsao

**Affiliations:** ^1^Cardiovascular Division, Department of Medicine, Beth Israel Deaconess Medical Center, Boston, MA, United States; ^2^Division of Cardiology, Department of Medicine, New York University Langone Health, New York, NY, United States

**Keywords:** aortic stiffness, pulse wave velocity, cardiovascular health, risk factors, biomarkers

## Abstract

Aortic stiffness (AoS) is a maladaptive response to hemodynamic stress and both modifiable and non-modifiable risk factors, and elevated AoS increases afterload for the heart. AoS is a non-invasive marker of cardiovascular health and metabolic dysfunction. Implementing AoS as a diagnostic tool is challenging as it increases with age and varies amongst races. AoS is associated with lifestyle factors such as alcohol and smoking, as well as hypertension and comorbid conditions including metabolic syndrome and its components. Multiple studies have investigated various biomarkers associated with increased AoS, and this area is of particular interest given that these markers can highlight pathophysiologic pathways and specific therapeutic targets in the future. These biomarkers include those involved in the inflammatory cascade, anti-aging genes, and the renin-angiotensin aldosterone system. In the future, targeting AoS rather than blood pressure itself may be the key to improving vascular health and outcomes. In this review, we will discuss the current understanding of AoS, measurement of AoS and the challenges in interpretation, associated biomarkers, and possible therapeutic avenues for modulation of AoS.

## Introduction

Aortic stiffness (AoS) is a measure of the elasticity of the blood vessel wall, and elevated AoS may result from and contribute to increased stress on the vessel walls. It is a non-invasive method of measuring maladaptive change and remodeling to aortic properties and is a promising marker of subclinical disease. Its measurement is based on principles of physics. The arterial tree has varying mechanical properties along its length, primarily determined by different contributions of collagen and elastin to its structure, in addition to varying degrees of modulation by smooth muscle. Pulse waves generated from pulsatile hemodynamics of the cardiac cycle travel down the large conduit arteries to the mid-sized arteries where they incur increased resistance due to branch points and increased arterial tone. The incident waves are then reflected back toward the central arteries from the periphery. The stiffness of the central conduit arteries determines the velocity with which the reflected waves return, with increased AoS resulting in more rapid propagation of reflected waves, determining the measured pulse wave velocity (PWV) (1). Pathologically increased AoS allows waves reflected from the periphery to return in phase with cardiac systole, augmenting central systolic pressure and increasing hemodynamic load on the left ventricle. AoS is able to capture a unique measure of central hemodynamics not reflected by simply the blood pressure alone, likely explaining the ability of carotid-femoral PWV (cfPWV) to serve as an independent predictor of cardiovascular outcomes. Further, the processes implicated in AoS, which include activation of oxidative stress pathways and inflammation may be reflective of underlying vascular risk (2).

The purpose of this review is to discuss the clinical implications of AoS, its measurements including PWV and augmentation index (AI), and the factors that contribute to and alter AoS. We will also review AoS involvement in disease processes as well as biomarkers involved in AoS. The goal is to gain a better understanding of AoS as a subclinical marker of chronic disease.

## Clinical Significance

Stiffening of the aorta is a marker of subclinical disease and has been demonstrated to precede the onset of hypertension in a longitudinally followed cohort (3). Earlier studies first implicated elevated PWV to be associated with atherosclerosis (4) and as a predictor of worse cardiovascular outcomes and mortality in high-risk conditions such as diabetes mellitus (DM), chronic kidney disease, and hypertension, as well as coronary artery disease post-myocardial infarction (5–7). AoS was later demonstrated in healthy community dwelling individuals to predict incident events including coronary disease, heart failure, stroke, and cardiovascular mortality independently of adjustment for cardiovascular risk factors (8–11). Further, there is evidence that AoS reflects the presence of composite end-organ damage and has been shown to have superior prognostic value to measurements of office and ambulatory systolic blood pressures in patients with advanced kidney disease (12).

The adverse outcomes related to elevated AoS suggested by the prior studies have been corroborated and further evaluated through meta-analyses. In such a 2010 study by Vlachopoulos et al. an increase in PWV of 1 m/s conferred an increased risk of cardiovascular events, cardiovascular mortality, and all-cause mortality (13). Moreover, a meta-analysis of over 17,000 participants showed that a 1-standard deviation difference in log-transformed cfPWV was associated with an increased risk of future cardiovascular events over 5 years even after adjusting for more traditional risk factors; furthermore, this same meta-analysis showed that using cfPWV in addition to traditional risk factors was able to reclassify patient risk for cardiovascular disease (CVD) for those who had an intermediate 10 year CVD risk (14). Therefore, by measuring AoS in patients, practitioners may be able to detect patients at risk for CVD at an early, subclinical stage. This early detection may provide the opportunity for early intervention, patient education on risk factors, and potentially help to decrease the incidence of overt disease.

## Measurement

Several modalities are available to measure AoS by PWV including recording the pulse waves by a tonometer transducer, standard blood pressure cuff, doppler ultrasound, and magnetic resonance imaging (MRI) (15). The transducer methods consist of placing a tonometer over the carotid and femoral arteries and monitoring an ECG signal for timing of the pressure waveforms. These methods have historically been the gold standard but can be a challenging learning curve for the operator. Thus, there has been increased interest in comparing the various AoS measurement methods to determine which is most accurate and easiest to implement. Pulse wave doppler ultrasound allows measurement of AoS without the need for a specific measurement device, is quicker, and has been shown to be comparable to transducer methods (16, 17). While blood pressure cuff measured cfPWV may be easier to acquire than the doppler approach, it often requires correction (18). MRI based techniques also offer promise due to their ability to directly and accurately visualize path-length and ability to quantify AoS in more proximal aortic segments but lack practicality (19).

**Figure 1:**
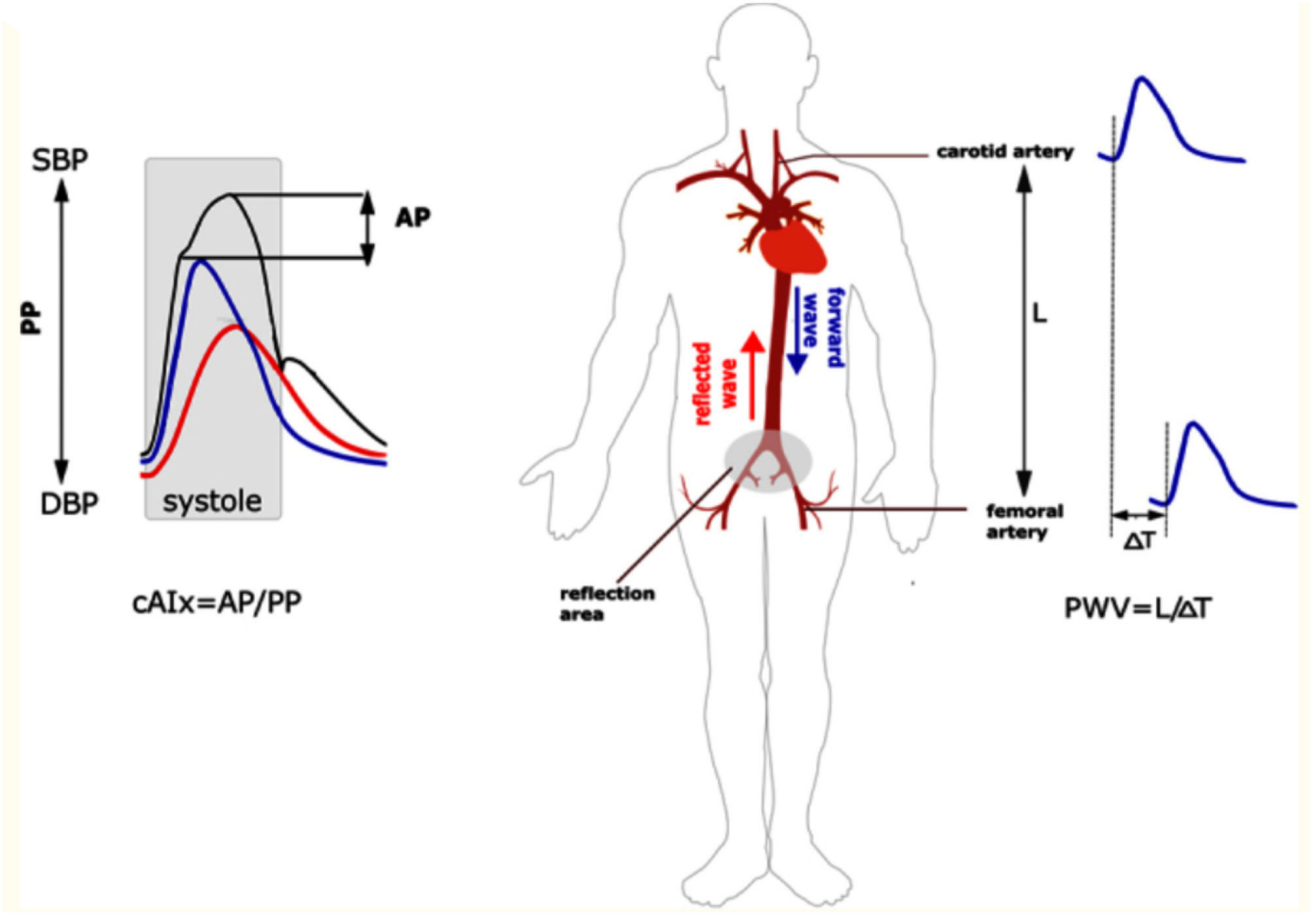
Measurement of augmentation index and cfPWV. On the left panel, the central augmentation index is calculated as the ratio of the augmentation pressure over the pulse pressure. On the right panel, the cfPWV is measured by evaluating waveforms at the common carotid and femoral artery. This is the foot-to-foot method as it measures the beginning of the waveform at each site. The velocity component is then calculated by measuring the distance between the two sites divided by the time it takes for the waveform to travel from site to site. AP, augmentation pressure; PP, pulse pressure; cAIx, central augmentation index; PWV, pulse wave velocity; SBP, systolic blood pressure; DBP, diastolic blood pressure. Reproduced from (21).

cfPWV is the gold standard measure of aortic wall stiffness (2). cfPWV is obtained via transcutaneous measurement of the pressure waveform at the common carotid artery and at the femoral artery by either probes or blood pressure cuffs; alternatively, this can be measured from Doppler or MRI flow waveforms (20). The distance between the two surface sites and the time delay between the waveforms is used to determine the velocity component (2). Figure 1 depicts how the cfPWV is calculated. It is important to note that blood pressure and PWV are closely intertwined with higher mean arterial pressures correlated with increased AoS (15, 22).

However, there are additional challenges to measuring cfPWV. The surface distance between carotid and femoral sites of measurement may not represent true arterial path-length, especially in patients with obesity. Therefore, proposed correction factor equations account for these systematic inaccuracies such as multiplying the distance from the carotid artery to the femoral artery by 0.8 (23). In addition, there are challenges with measuring pressure waveforms in obese patients and in controlling for existing atherosclerotic disease in vessels. Furthermore, conditions during time of measurements such as patient positioning, temperature in the room, and white coat hypertension can all confound the results (2). Brachial PWV methods also exist, but because of PWV amplification in peripheral arteries, it is considered a less reliable measure of central artery stiffness (24).

Augmentation index (AI) is another measurement of AoS (Figure 1). It is measured by dividing the augmentation pressure by the pulse pressure and multiplying by 100 to provide a number (percentage). AI is a stronger predictor of left ventricular mass reduction in response to lowering the blood pressure compared to other more conventional measures such as brachial blood pressure (25), and increased AI is independently associated with increased cardiovascular events in those undergoing percutaneous coronary interventions (26). Furthermore, more recent data has shown that higher augmentation index is associated with poor exercise capacity after heart transplant (27). However, AI is impacted by other factors such as age, systolic blood pressure, heart rate, left ventricular ejection time, and height to a greater extent than PWV (28, 29). Therefore, PWV, and in particular cfPWV, is used more often in trials.

## Normative Values and Impact of Demographics

Despite having known prognostic implications distinct from traditional cardiovascular risk factors, the clinical use of cfPWV has been limited due to lack of widespread use of population specific reference ranges. The 2007 ESC/ESH guidelines proposed a cut-off value of 12 m/s for elevated AoS based on clinical outcome data (30). Furthermore, multiple studies have sought to establish reference ranges for PWV. The Reference Values for Arterial Stiffness Collaboration Database was one of the first large-scale efforts to establish reference ranges for cfPWV in 16,867 European individuals across 13 centers (31). A subset of 11,092 individuals without prevalent CVD or use of anti-hypertensive or lipid-lowering medications were used to draw reference values presented in Table 1. However, a challenge with creating normative values is that experienced laboratories are needed for cfPWV measurement, and disparate measurement devices and methodologies can produce a variance in PWV affecting generalizability even within the same patient (32).

**Table 1:** Distribution of pulse wave velocity (PWV) values (m/s) in the reference value population (11,092 subject) according to age and blood pressure category.

**Age category (years)**	**Blood pressure category**				
	**Optimal**	**Normal**	**High Normal**	**Grade I HTN**	**Grade II/III HTN**
**PWV as mean**					
**(±2SD)**					
<30	6.1 (4.6–7.5)	6.6 (4.9–8.2)	6.8 (5.1–8.5)	7.4 (4.6–10.1)	7.7 (4.4–11.0)
30–39	6.6 (4.4–8.9)	6.8 (4.2–9.4)	7.1 (4.5–9.7)	7.3 (4.0–10.7)	8.2 (3.3–13.0)
40–49	7.0 (4.5–9.6)	7.5 (5.1–10.0)	7.9 (5.2–10.7)	8.6 (5.1–12.0)	9.8 (3.8–15.7)
50–59	7.6 (4.8–10.5)	8.4 (5.1–11.7)	8.8 (4.8–12.8)	9.6 (4.9–14.3)	10.5 (4.1–16.8)
60–69	9.1 (5.2–12.9)	9.7 (5.7–13.6)	10.3 (5.5–15.1)	11.1 (6.1–16.2)	12.2 (5.7–18.6)
≥70	10.4 (5.2–15.6)	11.7 (6.0–17.5)	11.8 (5.7–17.9)	12.9 (6.9–18.9)	14.0 (7.4–20.6)
**PWV as median**					
**(10th−90th percentile)**					
<30	6.0 (5.2–7.0)	6.4 (5.7–7.5)	6.7 (5.8–7.9)	7.2 (5.7–9.3)	7.6 (5.9–9.9)
30–39	6.5 (5.4–7.9)	6.7 (5.3–8.2)	7.0 (5.5–8.8)	7.2 (5.5–9.3)	7.6 (5.8–11.2)
40–49	6.8 (5.8–8.5)	7.4 (5.3–8.2)	7.7 (6.5–9.5)	8.1 (6.8–10.8)	9.2 (7.1–13.2)
50–59	7.5 (6.2–9.2)	8.1 (6.7–10.4)	8.4 (7.0–11.3)	9.2 (7.2–12.5)	9.7 (7.4–14.9)
60–69	8.7 (7.0–11.4)	9.3 (7.6–12.2)	9.8 (7.9–13.2)	10.7 (8.4–14.1)	12.0 (8.5–16.5)
≥70	10.1 (7.6–13.8)	11.1 (8.6–15.5)	11.2 (8.6–15.8)	12.7 (9.3–16.7)	13.5 (10.3–18.2)

*Modified from Reference Values for Arterial Stiffness (31)*.

*HTN, hypertension; SD, standard deviation*.

## Age, Sex, and Race

### Age

A rise in AoS with age has been well-described in large, diverse groups free of clinical CVD (31, 33–35). Central artery stiffness results in a reduced arterial reservoir effect, augmenting pressure during systole and diminishing it during diastole (36). This is thought to be one mechanism for the observed age-related increase in systolic blood pressure and decline in diastolic blood pressure, which lead to adverse ventricular and vascular hemodynamics, poor cardiac perfusion, and cardiac remodeling (37, 38).

Several mechanisms may contribute to age-related arteriosclerosis. Intrinsic remodeling of arteries has been demonstrated with increasing intima media thickness with age (39). Changes in the mechanical properties of the vascular media are also observed, with maladaptive remodeling with increased deposition of collagen (40). Age related arteriosclerosis that is independent of atherosclerosis is supported by the strong independent association between age and cfPWV that persists in those without aortic calcifications (41). The cumulative exposure to vascular risk factors including DM also contributes to increases in AoS with age (42).

### Sex

The relationship between sex and AoS is complex and varies with age. Whereas pre-pubescent females have higher PWV than pre-pubescent males, this difference is abrogated post-puberty as the average PWV in females decreases but PWV in males increases (43). In the Jackson Heart Study, while adult men were more likely to have elevated cfPWV in the overall cohort, women had steeper rise in both cfPWV and forward wave amplitude with age >60 (35). Brachial-ankle PWV (baPWV) has been shown to be similar in males and females until about age 50–60 years old, at which point there is a greater proportional increase in female baPWV (44). This accelerated increase in the baPWV around age 50–60, when females are post-menopausal, provides further evidence that there is a hormonal component to the sex differences in AoS. Furthermore, when corrected for age and blood pressure, middle aged females with metabolic syndrome had higher aortic PWV as compared to males, again supporting the role of sex the relationship of age with PWV (45).

The mechanism behind sex differences in AoS may be related to downstream effects of sex hormones. Men with acquired hypogonadism have higher PWV compared to normal men, and treatment with testosterone therapy helps to lower PWV, supporting a possible role for testosterone in lowering AoS (46). Indeed, in animal models, testosterone induces endothelium-independent vasodilation of arterial beds (47). Sex hormones in women also seem to play roles in modulating AoS. Decreases in estradiol with menopause are associated with a proinflammatory state, which may be a cause of elevated AoS in women after menopause (48). Furthermore, female sex steroids such as 17 beta estradiol and progesterone promote elastin deposition, and thus withdrawal following menopause may also contribute to increased AoS during this time period (49).

### Race

African Americans (AA) suffer a disproportionately increased risk of CVD, hypertension, and microvascular dysfunction compared to whites, highlighting the disparities in vascular morbidity and mortality (50–52). Data suggests that these differences may be driven by a difference in risk factor burden, sociodemographic factors including income, as well as intrinsic differences in mechanical properties of blood vessels and baseline AoS (53).

Differences in AoS between AA and whites have been observed in childhood. AA boys as young as 6–8 years old have elevated mean arterial pressure (MAP), intimal media thickness, and cfPWV compared to white boys (54). Sociodemographic factors including education, lower family income, and lower socioeconomic status were all associated with higher PWV (55). However, higher aortic PWV is seen in AA children compared to whites even after adjusting for age, sex, body mass index, mean arterial pressure, and socioeconomic status (56). This difference in AoS among children persists in adults. In the Multi-Ethnic Study of Atherosclerosis of multiple community cohorts, AA had a higher prevalence of hypertension and lower aortic distensibility (57). In the Dallas Heart Study, both AA and Hispanic individuals had greater aortic arch PWV independent of cardiovascular risk factors including mean arterial pressure, heart rate, DM, and smoking (58).

However, other studies suggest that there may be confounding variables that account for the differences in AoS among races. In the ELSA-Brasil study, investigators noted that AAs had a higher burden of hypertension, DM, and obesity compared to the other racial groups and had higher unadjusted cfPWV compared to browns and whites who were similar. However, after adjusting for characteristics including mean arterial pressure, age, waist circumference, heart rate, and fasting glucose, the inter-group differences were abrogated. The results of this study indicate that ~40% of the difference between cfPWV values could be explained by age and mean arterial pressure, suggesting less contribution by race itself to AoS. Though there may be a race-sex interaction in women, with AA and brown women having higher cfPWV than whites particularly in the highest quartiles of cfPWV, the strength of that relationship was much weaker than the effects of MAP and age (59).

Several mechanisms have been proposed to contribute to the differences of AoS among racial/ethnic groups. First, risk factor sensitivity may vary among different racial groups. For example, cfPWV progression is affected by risk factors such as diastolic blood pressure, glucose, and low-density lipoprotein cholesterol in AA women, while it was not in Caucasian women (60). Furthermore, it has been demonstrated that AAs living in the northern hemisphere likely suffer a greater burden of Vitamin D deficiency relative to white counterparts (61). Vitamin D has been proposed to improve vascular health by suppressing oxidative pathways and the sensitivity to renin-angiotensin-aldosterone system (RAAS) mediated remodeling. Vitamin D supplementation has been shown to decrease PWV in AAs with vitamin D deficiency (62). While the paucity of large-scale studies suggests the need for further research to determine the clinical utility of improving Vitamin D to improve vascular health, this modifiable risk factor creates a targetable treatment regimen for AAs.

Generalized endothelial dysfunction has also been posited as a mediator of progressive AoS in AAs compared to whites. Studies have demonstrated that AAs tend to have impaired nitric oxide signaling and thus more endothelial cell dysfunction at baseline and when compared to whites (63, 64). This impaired nitric oxide signaling in AAs compared to white Americans has been shown to be present even after adjusting for CVD risk factors, suggesting that impaired vascular function precedes incident disease (65). Whether additional intrinsic differences in the properties of vessels or unmeasured risk factors exist between racial/ethnic groups remains to be determined.

## Comorbidities Associated with AoS

### Hypertension

Hypertension demonstrates a very strong association with AoS compared to other cardiometabolic risk factors studied. A major shift has occurred regarding the understanding of directionality between hypertension on AoS and vice versa. The initial paradigm posited that arterial stress induced by elevated pressure and pulsatility-mediated breaks in elastin led to maladaptive remodeling by inducing inflammation (1, 66, 67). Both baseline blood pressure and blood pressure variability have been linked to increased vascular stiffness (68). Higher blood pressure variability is thought to promote vascular smooth muscle cell proliferation and atherosclerosis as well as increase oscillatory wall stress (69, 70). This increased variability may lead to increased AoS which in turn, with stiffer arteries, may increase blood pressure (71).

Clinical and experimental studies have demonstrated that the relationship between hypertension and AoS is interdependent (72–74). Elevated AoS preceding the development of overt hypertension has been demonstrated in population-based studies (3, 75). Additionally, Weisbrod et al. evaluated the temporal relationship between AoS and hypertension in a mouse model of diet induced obesity, demonstrating that AoS increased within 1 month while hypertension evolved in 5 months (76). Increased AoS and blood pressure were reversed with weight loss. Understanding this temporal relationship is of particular clinical significance, as AoS can be used as a marker for patients that are high risk to develop hypertension, prompting earlier risk factor modification and potential treatment.

### Metabolic Syndrome

While hypertension alone can increase AoS, metabolic syndrome is also associated with increased AoS. Metabolic syndrome is a constellation of disorders consisting of obesity, insulin resistance, hypertension, and dyslipidemia. Investigators from the Bogalusa Heart Study showed that even in asymptomatic, young (ages 24–44) subjects, baPWV rose with increasing number of components of the metabolic syndrome (77). Multiple other studies have shown that metabolic syndrome components were associated with elevated PWV (78–80). Investigators of the CRAVE study also showed that patients with both hypertension and dyslipidemia had a four-fold increase in the annual progression of cfPWV compared to controls (80). There is also evidence to suggest that resolving metabolic syndrome is associated with lower PWV compared to those with current metabolic syndrome (81).

### Diabetes Mellitus

Patients with DM are at a high risk for CVD (82, 83). Aortic PWV serves as an additional tool to help risk stratify patients as increased PWV has been shown to be associated with CVD in those with DM (84). An interesting dose dependent relationship between level of glucose dysregulation and elevation of cfPWV has also been described (85).

The pathogenesis of AoS in DM is likely to be mediated by the pro-inflammatory milieu generated by metabolic dysregulation and direct damage to the vascular wall. For example, high intake of advanced end glycation products, such as carboxy-methyl-lysine, have been associated with higher PWVs among those with DM (86). Furthermore, a trial of ALT-711, a non-enzymatic breaker of these products, decreased PWV in the elderly (87). Different genotypes of advanced end glycation products and their receptors have also been associated with increased blood pressure and AoS in patients with DM (88). Therefore, modulation of advanced end glycation products remains an interesting target to halt disease progression. Furthermore, increased glucose may lead to increased activity of RAAS and thus the detrimental consequences as described in the section on RAAS below (89).

## Lifestyle Risk Factors

The association of lifestyle risk factors with AoS detailed below are summarized in Table 2.

**Table 2:** Association of lifestyle risk factors with aortic stiffness.

	**Population studied**	**Exposure**	**Effect**
Diet	Adults 20–59 years of age	Salt consumption (varied)	An increase in urinary sodium excretion by >100 mmol over a 24-h period is associated with increased systolic pressures by 3–6 mm Hg and increased diastolic pressures by 0–3 mm Hg (90)
	11 adults aged 60 ± 2 years with elevated BP (139 ± 2 over 83 ± 2 mmHg)	Low sodium (77 ± 2 mmol/d) vs. normal sodium (144 ± 7 mmol/d)	Low sodium group with 17% reduction in aortic PWV compared to normal sodium (7 ± 0.40 vs. 8.43 ± 0.36 m/s, *p* = 0.001) (91)
Exercise	Endurance trained males age 69 ± 2.5 years	Fitness level: VO2 max at least 1 SD above age matched sedentary counterparts	26% decrease in Aortic PWV relative to peers their age (92)
	Pre-menopausal women aged 31 ± 1 years and post-menopausal women age 59 ± 2 years	6 ± 1 hour/week of endurance exercise	No significant difference in aortic PWV or AI between pre and post-menopausal women with exercise (suggesting age related increase in AoS is halted by exercise) (93)
	Systematic review/meta-analysis of 14 RCTs of adults with pre-hypertension and hypertension	Exercise types: aerobic/endurance, dynamic resistance, isometric resistance, combined exercise	Exercise significantly reduced PWV by 0.76 m/s (CI 1.05–0.47) (94)
Smoking	Healthy adults 33 ± 6 years of age	Acute: 5 min after smoking 1 cigarette	FMD % decreased from 13.5 ± 5 to 6.9 ± 4% (95)
	Adults 15–57 years of age	Chronic: 1–75 pack years	FMD 10 ± 3.3% (4–22%) in controls vs. 4 ± 3.9% (0–17%) in smokers; FMD is inversely related to the duration of smoking (96)
	Males 30–64 years of age	Non-smokers, former smokers, and current smokers	Men who quit smoking <1 year prior had elevated AI (β 3.94, SE 1.54, *p* = 0.011) similar to current smokers (β 4.39, SE 0.74, *p* <0.001) compared to non-smokers; those that quit 1– <10 years prior with AI similar to non-smokers (β 1.87, SE 0.94, *p* <0.047) (97)
E-cigarettes	Adults 30 ± 8 years of age	5 min of usage and 30 min of usage	Smoking over 5 min increased cfPWV by 0.19 m/s after 15 min; over 30 min increased cfPWV by 0.36 m/s (98)
Alcohol	Males 40–80 years of age	4–10, 11–21, and 22–58 drinks/week	Compared to those consuming 0–3 drinks per week; decreased cfPWV by 0.77 m/s (4–10 drinks), 0.57 m/s (11–21 drinks), 0.14 m/s (22–58 drinks) (99)
	Post-menopausal women 50–74 years of age	1–3, 4–9, 10–14, and 15–35 drinks/week	Compared to non-drinkers: those consuming 1–3, 4–9, 10–14, and 15–35 drinks/week had the following difference in mean cfPWV 0.044 (95% CI −0.47–0.56), −0.085 (95% CI −0.59–0.43), −0.869 (95% CI −1.44–0.29), and −0.225 (95% CI −0.98–0.53) m/s (100)

*AI, augmentation index; AoS, aortic stiffness; β, beta; BP, blood pressure; cfPWV, carotid-femoral pulse wave velocity; FMD, flow-mediated dilation; PWV, pulse wave velocity; RCT; randomized control trial; SD, standard deviation; SE, standard error*.

### Alcohol

Much of the evidence for the association of alcohol with AoS is data derived from self-reported alcohol consumption in cross-sectional epidemiology studies. Interestingly, evidence suggests there may be a J shaped relationship of alcohol use to central aortic hemodynamics, with more favorable measures among those with light to moderate consumption compared with negligible and heavy drinkers. In young individuals, those who reported light alcohol consumption, 2–6 drinks per week, had lower central blood pressure than those who drank lower or greater amounts (101). Similar findings have been reported in middle aged to older adults. In men aged 40–80 years old, those who drank moderate to large amounts of 4–10 and 11–21 drinks per week had lower PWV than those who drank more or less than these groups (99). Additionally, in post-menopausal women aged 50–74 years, moderate alcohol intake was inversely related to PWV (100). Furthermore, in controlled experiments, alcohol ingestion appears to acutely decrease AoS. Even drinking 200 or 350 cc of beer leads to decreased baPWV and cfPWV compared to controls (102). There are many proposed mechanisms as to why low doses of alcohol can be beneficial to the heart such as by increasing HDL, insulin sensitivity, and decreasing oxidative stress (103). More acutely, small amounts of alcohol may decrease PWV through alcohol induced increases in nitric oxide (104). Ultimately, future prospective studies will shed light on the ideal alcohol consumption with respect to long-term outcomes and recommended exposure for vascular health.

### Smoking

Smoking is a major modifiable risk factor for CVD (105). One cigarette causes acute increases in brachial and aortic blood pressure, arterial wave reflection, and AoS (106). Even passive smoking has been shown to worsen the elasticity of the aorta (107). Cigarette smoking has been shown to have a dose-response relationship to elevated PWV, which is only reversed after prolonged smoking cessation >10 years (108). There is also evidence to suggest that e-cigarettes are detrimental to AoS (98). The effect of cigarettes on AoS may be due to endothelial cell damage and subsequent impaired vasodilatory capacity (95, 96). Additional mechanisms appear to be an increase in cholesterol, increased vascular remodeling and arterial calcification, increased vascular tone, and oxidative stress/inflammation (109).

### Diet and Physical Activity

There is a growing body of evidence that lifestyle habits including smoking cessation, diet modification, and exercise/weight loss can reverse AoS (110). It is well-supported that high salt intake leads to higher blood pressures (90) and that reduction in salt intake leads to lower blood pressures (111). Low salt diets similarly have been associated with lower PWV independent of blood pressure (112). Furthermore, in men, over a period of 17.8 years, higher consumption of saturated fatty acids was associated with higher cfPWV and higher consumption of poly-unsaturated fatty acids was associated with lower cfPWV (113). Greater dairy consumption, particularly in those with DM, as well as increased intake of vegetables has also been associated with lower AoS (114–116).

Physical activity leads to lower central PWV and age-related increases in PWV can be mitigated by exercise in both men and women (92, 93). The Baltimore Longitudinal Study of Aging rigorously phenotyped adults and measured VO2 max in adults aged 21–96 years of age. These investigators demonstrated that with greater age in the entire cohort, augmentation index and aortic PWV increased out of proportion to the blood pressure increase. However, these measures of AoS were lower in endurance trained male athletes (defined by a VO2 max 1 standard deviation above their age matched non-trained controls), compared with sedentary individuals (defined as less than at least 20 min of aerobic exercise three times weekly) of similar age (92). Similarly, while sedentary post-menopausal women have higher augmentation index and PWV than comparable pre-menopausal women, these measures of AoS were similar in both pre- and post-menopausal active women who were physically active (performed endurance training, actively competing in running races, with average exercise of 6 +/– 1 hour of activity per week) (93). The effect of exercise on reducing AoS is thought to relate to exercise induced changes in vessel wall stress, a reduction in vasoconstrictors and ultimately vasodilation via increased nitric oxide activity (117, 118). These studies add to the growing body of evidence that improved lifestyle modifications could make a large impact on the development and progression of disease.

## Biomarkers Illuminating Pathophysiology and Therapeutics

Given the association of AoS with adverse outcomes, serum biomarkers that correlate with AoS allow further insight into mechanisms of AoS, non-invasive detection and monitoring of AoS, and may highlight therapeutic targets. In this section, we will discuss key serum biomarkers that modulate AoS, and the associated evidence for therapies targeting these pathways.

### Inflammatory Biomarkers

The presence of chronic inflammatory and infectious conditions is associated with elevated AoS. In patients with systemic lupus erythematosus, cfPWV was shown to be elevated even when traditional risk stratification categorized patients into low risk for CVD (119). Furthermore, higher aortic PWV has been seen in patients with inflammatory bowel disease and has been associated with disease duration (120). Many other inflammatory conditions have been associated with increased AoS such as rheumatoid arthritis (121, 122), psoriatic arthritis (123, 124), and Sjogren's syndrome (125, 126). The increase in AoS in those with autoimmune disorders and chronic inflammatory diseases is independent of more traditional risk factors and related to disease duration and the elevation in inflammatory markers, suggesting inflammation as a key player in this pathology (127).

Multiple inflammatory biomarkers have been associated with AoS. A prospective study that followed middle-aged Japanese men without hypertension for 9 years demonstrated that sustained elevations in serum C-reactive protein (CRP) were associated with a longitudinal increase in baPWV. Higher baPWV was in turn associated with higher blood pressures during follow-up (128). The accelerated vascular disease in this cohort at relatively low vascular risk suggests that chronic inflammation may contribute to progressive vascular stiffness and dysfunction. Though CRP is associated with several cardiovascular risk factors, models adjusting for these demonstrated a persistent linear association between CRP and AoS in the population-based Rotterdam Study (129). A potential mechanism may lie in endothelial dysfunction: in men with coronary artery disease with forearm blood flow response studied with venous occlusion plethysmography, CRP levels were associated with blunted endothelial vasodilator capacity in models including risk factors (130). Additionally, normalization of CRP levels was associated with improved blood flow response in these individuals. IL-6 is another inflammatory cytokine that has been shown to be associated with cfPWV in individuals with hypertension (131). Furthermore, there is research establishing a link between polymorphisms on IL-6 with increased cfPWV (132). These studies suggest that inflammation is associated with AoS but more studies are needed to fully elucidate the mechanistic relationships.

The relationship between inflammatory states and CVD has been further elucidated by studies that have examined the effect of treatment of inflammatory diseases. Patients with rheumatoid arthritis treated with anti-tumor necrosis factor-a agents have shown significant declines in cfPWV after treatment (133). Furthermore, statins have been shown to decrease AoS in patients with inflammatory joint diseases, suggesting that controlling inflammation and possibly lowering lipids is beneficial in this population (134).

### Klotho and Sirtuin-1

Klotho is predominantly expressed in the kidney and has been described as an anti-aging gene (135). When mice are deficient in Klotho, they have decreased lifespan and calcifications of multiple organs. Haplodeficiency of Klotho in mice leads to increased PWV and hypertension (136, 137). The association of Klotho levels with AoS has also been demonstrated in patients with chronic kidney disease (CKD) (138). Klotho appears to directly regulate SIRT1, a gene encoding a NAD+ dependent-deacetylase with anti-inflammatory and anti-oxidant effects and importance in endothelial cell function (139, 140). Klotho haplodeficiency downregulates SIRT1 in arterial endothelial and smooth muscle cells, with associated increased arterial wall collagen deposition and elastin fragmentation, both of which explain the association with AoS (137). Zhou et al. demonstrated that CYP11B2, a rate-limiting enzyme in aldosterone synthesis, is up-regulated in Klotho deficiency, and that treatment with eplerenone reversed increased AoS (141). Thus, another mechanism by which Klotho deficiency may mediate increased AoS is through the aldosterone pathway.

The interaction between Klotho and SIRT1 has illuminated a number of possible targets for therapies that modulate pro-oxidant and pro-inflammatory pathways. Further, improved calcium and phosphate homeostasis may be of increased importance in CKD patients where impaired calcium homeostasis and a pro-inflammatory milieu may accelerate vascular dysfunction. Thus, understanding these mechanisms provides opportunities for possible therapeutic interventions.

### RAAS

The role of RAAS in the progression of AoS is supported by observational studies, clinical studies relating to modulation with therapeutics, biochemical studies demonstrating involvement in vascular remodeling, and mapping of related gene loci.

RAAS-associated AoS is proposed to be due to aldosterone and angiotensin II increased inflammation as well as vasoconstriction from activation of angiotensin I receptors and mineralocorticoid receptors (142). Aldosterone has been shown to be involved in many pathologic processes such as increased insulin resistance, increased oxidative stress, and increased inflammation (89). In multivariable adjusted models, serum aldosterone is linearly associated with PWV in hypertensive patients (143). The importance of RAAS is further highlighted by multiple studies that demonstrate a positive association between cfPWV and polymorphisms in the angiotensin II type 1 receptor (144, 145), angiotensin converting enzyme gene (146, 147) as well as in the aldosterone gene (148). Polymorphisms in RAAS may thus contribute to the highly heritable traits of AoS and blood pressure (149). Additional future work may determine the appropriate application of genetic testing to guide detection and management of AoS.

With respect to therapies, inhibiting aldosterone with spironolactone has been shown to decrease collagen density and thus AoS (150). London et al. demonstrated that central systolic blood pressure was decreased to a greater extent with perindopril/indapamide treatment compared to treatment with atenolol, implying a distinct role of RAAS modulation in central hemodynamics (151). This data on the role of RAAS inhibition in AoS may be useful to consider for physicians choosing an anti-hypertensive medication. When compared with atenolol, eplerenone has been shown to decrease AoS, decrease the collagen to elastin ratio, and decrease concentrations of inflammatory markers including MCP-1, basic fibroblast growth factor, and interleukin-10 (152). Furthermore, when comparing atenolol, nebivolol, aliskiren, and quinapril, the RAAS modulating agents demonstrated continued reductions of cfPWV, possibly implicating arterial remodeling rather than modulation of hemodynamics alone (153). Lastly, non-pharmacologic augmentation to the RAAS system is also important to consider. Decreased salt intake has been shown to decrease AoS independent of blood pressure reductions that may be mediated through RAAS modulation (154).

In addition to the above, other general biomarkers associated with AoS are presented in Table 3.

**Table 3:** Association of serum biomarkers with aortic stiffness.

**Biomarker**	**Clinical relevance**	**Association with aortic stiffness**
**Key biomarkers with independent association with AoS**		
Inflammatory biomarkers	•The presence of conditions like SLE (155), IBD (156), psoriasis (157), and HIV (158) are linked with high higher risk of CVD	•Elevated PWV in IBD patients (120) •Elevated carotid AI and PWV in SLE patients (159)
CRP	•Associated with insulin resistance (160), carotid intima-media thickness and markers of atherosclerosis (161)	•Sustained elevation in serum CRP correlated with increased baPWV and BP in middle aged Japanese men (128) •In Chinese general population baseline hs-CRP associated with baPWV (162)
Klotho	•Klotho levels lower in those with renovascular hypertension and essential hypertension compared to healthy controls (163) •Klotho levels lower in those with significant coronary artery disease (164)	•Haplodeficiency in Klotho in mice led to increased AoS (136, 137)
Aldosterone	•Increases insulin resistance, oxidative stress, inflammation (89) •Promotes vascular calcification (165)	•Associated with increased PWV (143) •Fibronectin accumulation (166)
**Other biomarkers associated with AoS**		
Adipocyte-Fatty-Acid-Binding protein (A-FABP)	•Elevated levels have been associated with endothelial dysfunction in patients with type 2 diabetes (167) •Elevated levels associated with diastolic dysfunction (168) and cardiovascular death (169)	•In patients with hypertension and metabolic syndrome, increased levels of A-FABP associated with increased cfPWV (170) •A-FABP levels positively correlated with cfPWV in patients with type 2 diabetes (171)
Leptin	•Leptin levels predicted ischemic heart disease in patients with type 2 diabetes (172) •Patients with coronary artery disease have higher levels of serum leptin (173)	•Higher leptin levels associated with increased cfPWV in patients with kidney transplants (174) and in geriatric patients on dialysis (175) •Meta-analysis demonstrated leptin is positively associated with cfPWV (176)
Natriuretic peptides	•Released in response to ventricular hypertrophy, inflammation, and fibrosis (177) •Predictor for heart failure or death in patients with an acute MI (178, 179)	•AoS is associated with NT-proBNP level and MR-proANP months after MI (180, 181)
Parathyroid hormone	•Parathyroid hormone is associated with atherosclerosis (182)	•Patients with mild hyperparathyroidism had increased cfPWV which then decreased after a thyroidectomy (183) •cfPWV increased independently with parathyroid hormone in Chinese patients with untreated hypertension (184)
Resistin	•Increased resistin associated with increased risk of heart failure, coronary heart disease, CVD (185)	•High levels of resistin associated with increased cfPWV in sample with high prevalence of untreated hypertension/obesity (186) •Serum resistin is an independent predictor of cfPWV in patients with coronary artery disease (187)
Uric Acid	•High levels of uric acid associated with acute myocardial infarction (188) cardiovascular events (189, 190) stroke (190)	•Association between higher uric acid and cfPWV in men after adjustment for confounders (191) •Overall positive association between uric acid and cfPWV at adjusted analysis in both males and females (192) •Serum uric acid is independently associated with cfPWV in post-menopausal women (193) •Significant association between uric acid cf PWV and carotid radial PWV in young Caucasian population (194)

*AI, augmentation index; AoS, aortic stiffness; ba-PWV, brachial-ankle pulse wave velocity; CVD, cardiovascular disease; HIV, human immunodeficiency virus; hs-CRP, high sensitivity CRP; IBD, Inflammatory Bowel Disease; MI, myocardial infarction; PWV, pulse wave velocity; SLE, Systemic lupus erythematosus*.

## Generalizability and Future Directions

Despite data illuminating pathways important in AoS pathophysiology and the promising data for their modulation, there has been a paucity of data in this field. Controlled trials thus far have been of relatively small size with short duration, with possibly insufficient follow up time to adequately assess for aortic remodeling and change in AoS (195). However, encouraging data on the prognostic impact of PWV continues to emerge. In the past 2 years, a *post-hoc* analysis of 8,450 patients in the Systolic Blood Pressure Intervention Trial (SPRINT) demonstrated that reductions in PWV after 1 year of anti-hypertensive therapy were associated with 42% lower risk of death compared to individuals who did not have reductions in PWV, independent of Framingham Risk Score and blood pressure (196). Additionally, an innovative experiment performed on mice aortas *ex vivo* used a synthetic peptide targeted to a cytoskeletal protein known to be associated with AoS in human genome wide association studies (197). This study illustrated the proof of concept that such decoy peptides decreased cfPWV, illustrating that approaches targeted to AoS rather than blood pressure *per se*, may be able to be applied in the future. Ultimately, larger therapeutic trials that target AoS and demonstrate improved outcomes are needed to establish widespread clinical utility of AoS assessment and treatment.

## Conclusion

AoS is a precursor to hypertension and an accepted risk factor for CVD independent of blood pressure. Despite its demonstrated prognostic value, thus far broad clinical applicability has been limited by measurement variation in multiple methodologies illustrated, lack of age and blood pressure specific reference values applicable to all populations, and effective therapeutics targeting AoS. AoS may be addressed indirectly through treating several lifestyle risk factors and associated comorbidities. Continued research will help to add to the illustrated biologic pathways of AoS. In the future, novel approaches and applications of existing drugs to specifically target pathways involved in modulating AoS may provide further support to its broader assessment and treatment to improve cardiovascular outcomes.

## Author Contributions

RA: drafted manuscript, manuscript editing, and figure copyright permissions. RM: drafted manuscript. CT: manuscript concept and editing. All authors contributed to the article and approved the submitted version.

## Funding

This work was partially supported by NHLBI R03HL145195 and R01HL155717 (to CT).

## Conflict of Interest

The authors declare that the research was conducted in the absence of any commercial or financial relationships that could be construed as a potential conflict of interest.

## Publisher's Note

All claims expressed in this article are solely those of the authors and do not necessarily represent those of their affiliated organizations, or those of the publisher, the editors and the reviewers. Any product that may be evaluated in this article, or claim that may be made by its manufacturer, is not guaranteed or endorsed by the publisher.
